# Multimodal medical image fusion algorithm based on pulse coupled neural networks and nonsubsampled contourlet transform

**DOI:** 10.1007/s11517-022-02697-8

**Published:** 2022-11-07

**Authors:** Sa.I. Ibrahim, M. A. Makhlouf, Gh.S. El-Tawel

**Affiliations:** 1 Information Systems Department, Faculty of Computers and Informatics, Suez Canal University, Ismailia, Egypt; 2 Computer Science Department, Faculty Of Computers and Informatics, Suez Canal University, Ismailia, Egypt

**Keywords:** Medical image fusion, Pulse coupled neural networks, Nonsubsampled contourlet transform, Computed tomography, Magnetic resonance image

## Abstract

**Abstract:**

Combining two medical images from different modalities is more helpful for using the resulting image in the healthcare field. Medical image fusion means combining two or more images coming from multiple sensors. This technology obtains an output image that presents more effective and useful information from two images. This paper proposes a multi-modal medical image fusion algorithm based on the nonsubsampled contourlet transform (NSCT) and pulse coupled neural networks (PCNN) methods. The input images are decomposed using the NSCT method into low- and high-frequency subbands. The PCNN is a fusion rule for integrating both low- and high-frequency subbands. The inverse of the NSCT method is to reconstruct the fused image. The results of medical image fusion help doctors with disease diagnosis and patient treatment. The proposed algorithm is tested on six groups of multi-modal medical images using 100 pairs of input images. The proposed algorithm is compared with eight fusion methods. We evaluate the performance of the proposed algorithm using the fusion metrics: peak signal to noise ratio (PSNR), mutual information (MI), entropy (EN), weighted edge information (Q$^{AB/F}$), nonlinear correlation information entropy (Q$_{ncie}$), standard deviation (SD), and average gradient (AG). Experimental results show that the proposed algorithm can perform better than other medical image fusion methods and achieve promising results.

**Graphical abstract:**

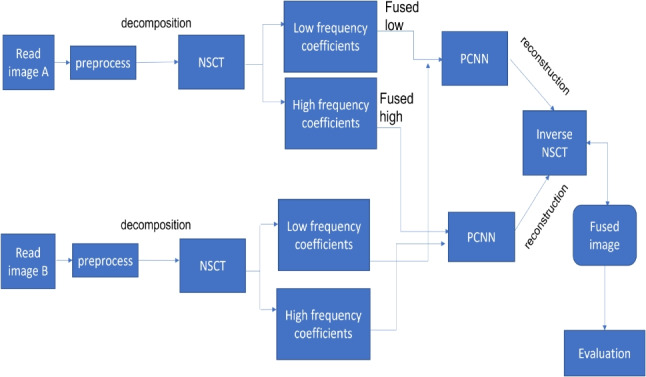

## Introduction

Medical images play an essential role in healthcare applications like disease diagnosis and patient treatment [9]. These images are capturing from different modalities such as magnetic resonance imaging (MRI), computed tomography (CT), positron emission tomography (PET), and single-photon emission computed tomography (SPECT).

All of these images are spot on different organ information. The CT images are used to visualize bone structure, and the MR images are used to visualize the internal or soft structures of the organ where the CT image is more accurate than the MRI image. On the other hand, PET and SPECT images provide metabolic or functional information in low resolution for the organ and are more accurate in tumor detection [12, 14, 25]. Table 1 describes the advantages and disadvantages of multimodality medical image.

**Table 1:** Multimodal medical image examples [9, 12, 25]

Modal	Example	Advantage	Disadvantage
CT		Scan in low time, less distortion, higher resolution,	poor contrast for soft tissue
		and more accurate than MRI	
MRI		high resolution in the spatial domain, more safe	not accurate, difficult for dealing with
		for pregnant women and preview anatomical details	movement organs like mouth tumors,
			and low sensitivity
PET		high sensitivity and accuracy in tumor detection	low resolution and high cost
SPECT		accurate in tumor detection and high sensitivity	low resolution, low image quality,
			high cost, and blur effects

**Fig. 1:**
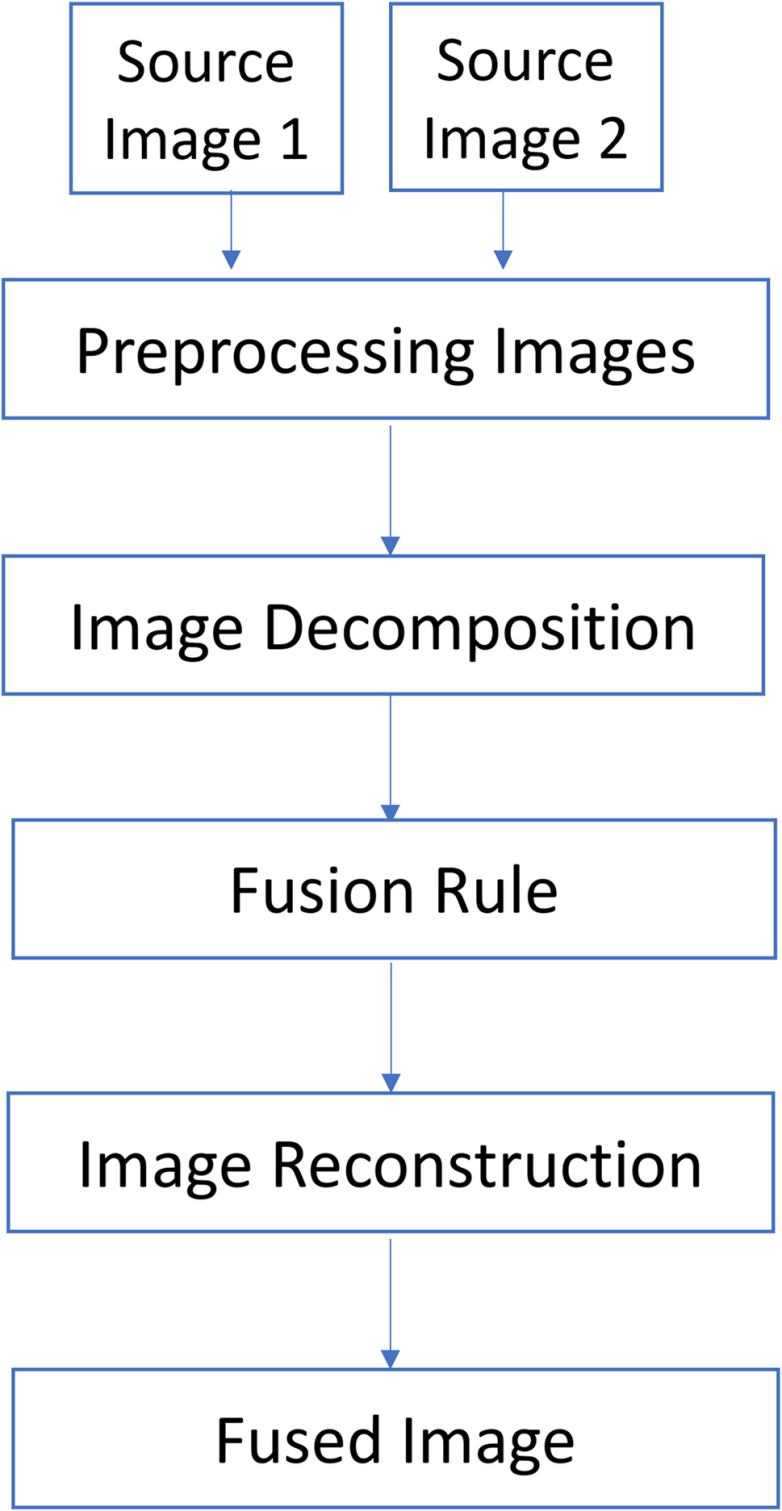
Image fusion process

There are three categories of image fusion like pixel-level fusion methods, feature-level fusion methods, and decision-level fusion methods [22]. Pixel-level fusion seeks to obtain the fused image by integrating the pixel information of input images. Feature-level fusion extracted the meaningful features from the input images and merged them in a single vector [6]. Pixel-level fusion is performed in either the spatial or transform domain. It is widely used in medical image fusion.

The spatial domain image fusion techniques focused on the input image pixels. The main advantage of this domain is low computational time. On the other hand, it introduces spatial distortion and produces color distortion and low contrast images [20]. The common examples of spatial domain-based image fusion methods are the principal component analysis (PCA) method, average fusion method, weighted average fusion method, minimum fusion method, and maximum fusion method.

Transform domain image fusion techniques aim to get low- and high-frequency coefficients by transforming the input images into the frequency domain rather than a spatial domain. It is more accurate and efficient than spatial domain methods. The advantages of the transform domain method are avoiding distortion and dealing with multiple resolution images (Fig. 1).

The common medical image fusion methods in the transform domain are based on multiscale transform (MST) to obtain a good result. The MST fusion methods performed in three steps are decomposition, fusion, and reconstruction [14, 30]. The common MST methods are Laplacian pyramid (LP) [2, 4], discrete wavelet transform (DWT) [21], nonsubsampled shearlet transform (NSST) [23], convolutional neural networks (CNN) [14], and NSCT [31]. The basic image fusion process is described in these steps:

- Image decomposition: convert the source images into an MST domain.
- Fusion rule: apply the fusion rule to merge the transformed coefficients.
- Image reconstruction: apply the inverse transform to reconstruct the fused image.

### Motivations

Medical images are accurate images that require massive effort to clean and prepare for usage. These images face two challenges. To begin, collect medical images in high resolution. Second, create a good image fusion algorithm that preserves all the salient features in the source images.

The main motivations for this paper are choosing the most effective method for combining several source images with the following characteristics: high efficiency, high spatial resolution preservation, and low color distortion using the PCNN in the NSCT domain to aid doctors in accurately diagnosing diseases. It also creates a new accurate fused image with more detailed information than the input images.

### Contribution

Our proposed medical image fusion method uses the NSCT features, including multi-scale, shift-invariance, and multi-directional properties, along with the PCNN to gain high fusion performance and capture the subtle differences and fine details present in the source medical images. The proposed method enhances the output fused image’s high contrast, clarity, and information content.

The main contribution of this paper is to create a high-performance fusion algorithm to detect whole brain regions from different multimodality medical images.

In summary, we propose a fused algorithm based on the PCNN method for multimodality medical images in the NSCT domain to improve the fused image quality to aid doctors in disease diagnosis. The rest of the paper is organized as follows. Section 2 focuses on some previous works. In Section 3 presents the proposed algorithm used in this paper. The experimental results and performance evaluation are discussed in Section 5. Finally, We conclude and summarize whole the paper in Section 6.

## Related work

Researchers presented multiple medical image fusion methods. All of these methods are tested and achieved good results. In this section, we preview and analyze some of this research.

This paper designs an effective CT and MR image fusion method [6]. In this work, the NSCT decomposes the source images. A maximum entropy of the square of the coefficients within a local window merged the low-frequency sub-bands. Maximum-weighted sum-modified Laplacian merged the high-frequency sub-bands. Finally, the inverse NSCT creates the fused image. We evaluate the proposed method using the CT and MR images for different cases and then compare the results with the other conventional image fusion methods. Both visual analysis and quantitative evaluation of experimental results show the superiority of the proposed algorithm over other methods.

Nazrudeen et al. [19] proposed a medical image fusion method based on NSCT. In this paper, the fusion process can be stated as follows: apply input image decomposition using the NSCT domain into low and high-frequency subbands. Apply phase congruency and directive contrast methods as a fusion rule. To produce the fused image, use the inverse NSCT method. The proposed method tested on Alzheimer, stroke, and tumor data, using CT and MRI datasets as input images. Whole experiments are applied in the MATLAB toolbox. Results are evaluated using PSNR (peak signal to noise ratio) and RMSE (root mean square error) measures. The proposed method is compared with classical fusion methods and produces higher image performance than other compared methods.

Manker et al. [18] proposed the NSCT fusion method and pixel-level fusion to fuse multimodal medical images. In this paper, use CT and MRI as input images. The input images are decomposed by NSCT transformation. The Gabor filter bank is applied on low-frequency coefficients and used the gradient fusion method on high-frequency coefficients. The inverse of NSCT transformation is applied to the resulting image to obtain the fused image. The results were evaluated by using common metrics such as entropy, PSNR, correlation coefficient, and MSE (mean square error).

Gomathi et al. [7] presented the NSCT method to fuse medical images. In this paper, the input images are decomposed into low-frequency and high-frequency coefficients by using the NSCT method. The maximum local mean and the maximum local variance are two fusion rules used. The maximum local mean method is applied on low-frequency coefficients and the maximum local variance method for high-frequency coefficients. The inverse of the NSCT method is to reconstruct the fused image. The presented method is tested on CT, MRI, and PET images using MATLAB R2010a. The common quality metrics such as entropy, standard deviation, mean, and edge-based similarity measure $(Q^{AB/F})$ results declare that the applied method is better than compared methods.

Tain et al. [24] presented an improved PCNN (IPCNN) based on the NSCT domain. In this paper, apply the NSCT method to decompose input images into subbands. Next, apply the IPCNN method as a fusion rule into the merged low and high subbands. Finally, perform the inverse NSCT to get the fused image. The results were evaluated by using common metrics such as entropy, mutual information, and weighted edge information. The experiment results show that the proposed method is better than other compared methods to fused medical images.

Xia et al. [28] presented a combination of sparse representation, NSCT transform, and PCNN method to fuse medical images. This combination aims to solve the NSCT problem in a low subband coefficient that is not sparse. The proposed fusion strategy is performed in three steps. First, decompose the input image using NSCT transform. Second, use the sparse representation and PCNN algorithm as the fusion rules respectively on low subbands and high subbands. Finally, use the NSCT inverse to produce the fused image. The result was evaluated by seven metrics such as standard deviation (SD), information entropy (IE), average gradient (AG), spatial frequency (SF), mutual information (MI), and edge information delivery factor, and structural similarity model (SSIM). The result shows the fused image with higher performance and better contrast than other compared methods.

Zhu et al. [32] proposed a new multimodal medical image fusion strategy based on NSCT transform and also used phase congruency and local Laplacian energy algorithms. The procedure of the proposed method is performed in three main steps. First, apply the NSCT method to decompose the input images into both lowpass and highpass subbands. Then, apply the local Laplacian energy fusion rule on the lowpass subbands and use the phase congruency fusion rule on the highpass subbands. Finally, apply the inverse NSCT transformation on the merged result from both lowpass and highpass subbands to produce the final fused image. The experiment results show that the performed method obtained high-performance fusion result with low computational time. The main defect of this method is not good to fused PET-MRI images.

## Material and methods

### Non subsampled contourlet transform (NSCT)

The contourlet transform (CT) method is used in image processing especially in geometric transformations and produces good results in this field [7]. The main problem of the CT method is a shift variant caused by down- and upsampling [32]. The NSCT method is a shift-invariant, multi-directional transform, and multi-scale image representation that depends on the CT theory and is applied by a` trous algorithm.

**Fig. 2:**
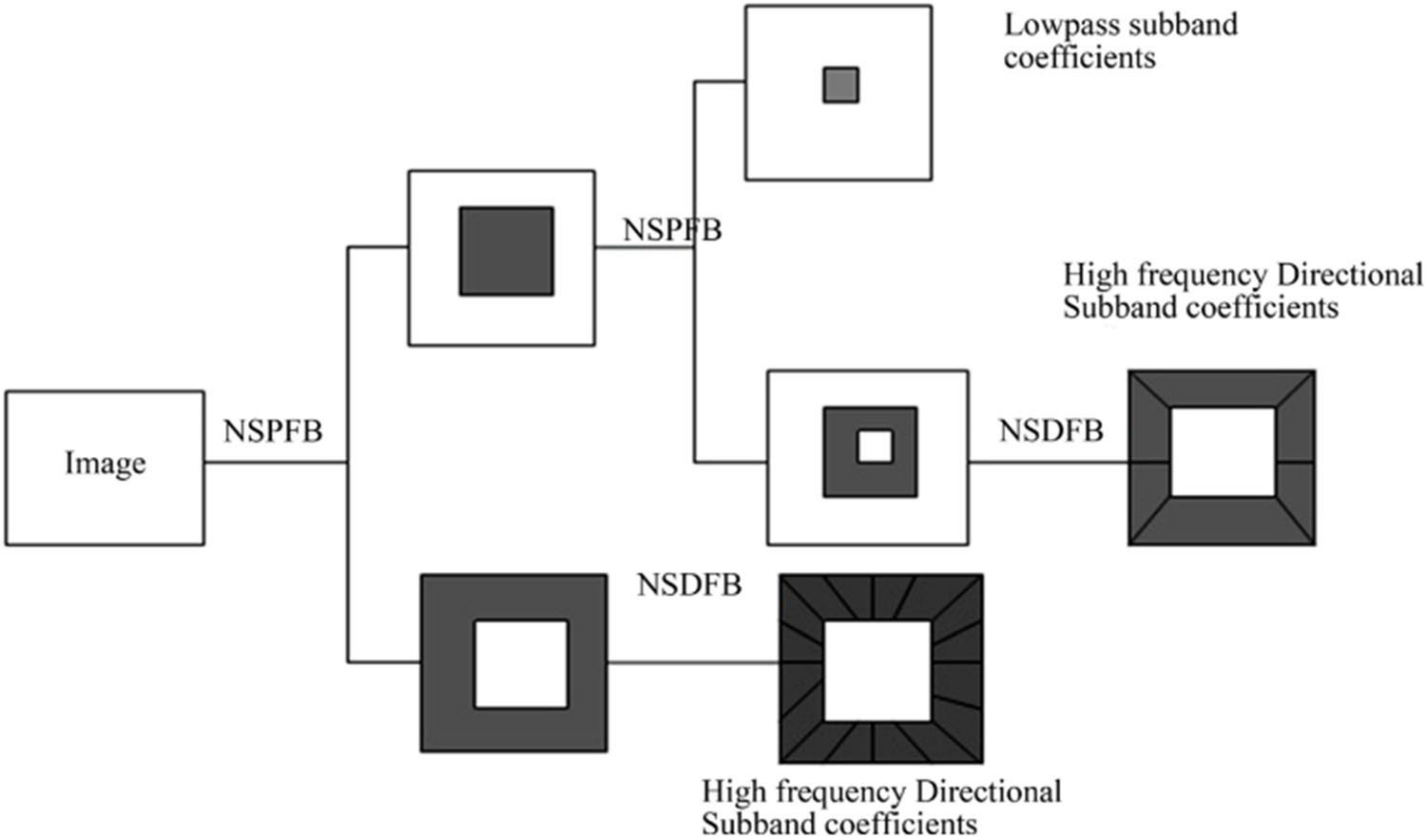
The NSCT image decomposition process [7]

This method is achieved by applying two basic stages: the nonsubsampled pyramid filter bank (NSP or NSPFB) and the nonsubsampled directional filter bank (NSDFB) [18, 19, 32]. The multiscale and multi-directional transform is ensured by both NSPFB and NSDFB filters. The image decomposition steps using the NSCT method are described as in Fig. 2.

The main steps of basic NSCT transform in medical image fusion are stated as in the following Algorithm 1

**Figure:**
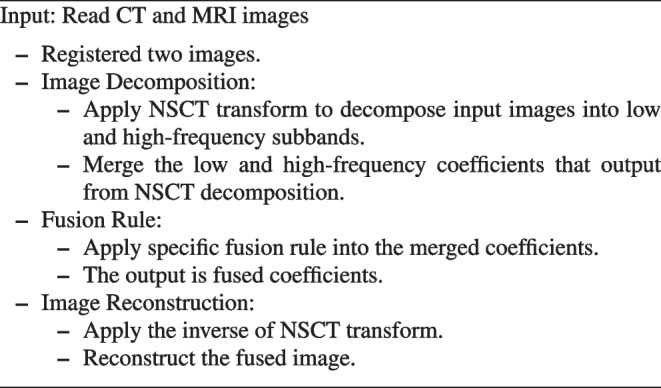
**Algorithm 1** The basic NSCT for medical image fusion algorithm.

#### Nonsubsampled pyramid filter bank (NSPFB)

The NSPFB consists of a two-channel filter bank without downsamplers and upsamplers [6, 32]. This filter bank aims to achieve multiscale decomposition for input images into the low-pass and high-pass subbands. Each NSPFB decomposition level aims to obtain both low- and high-pass frequency images. Then, the low-frequency image is decomposed iteratively by NSPFB. The result is M+1 sub-images, where M represents high-frequency images, and 1 represents the low-frequency image [7, 32].

#### Nonsubsampled directional filter bank (NSDFB)

NSDFB is a nonsubsampled filter bank consisting of two channels that are obtained by merging the directional fan filter-banks [7]. This filter bank aims to decompose the high-frequency images resulted from NSP decomposition to result at the directional sub-images, where the size of the source image and directional sub-images are the same. The NSDFB ensures the NSCT produces accurate directional detail information and multi-directional feature [7, 32].

### Pulse coupled neural networks (PCNN)

**Fig. 3:**
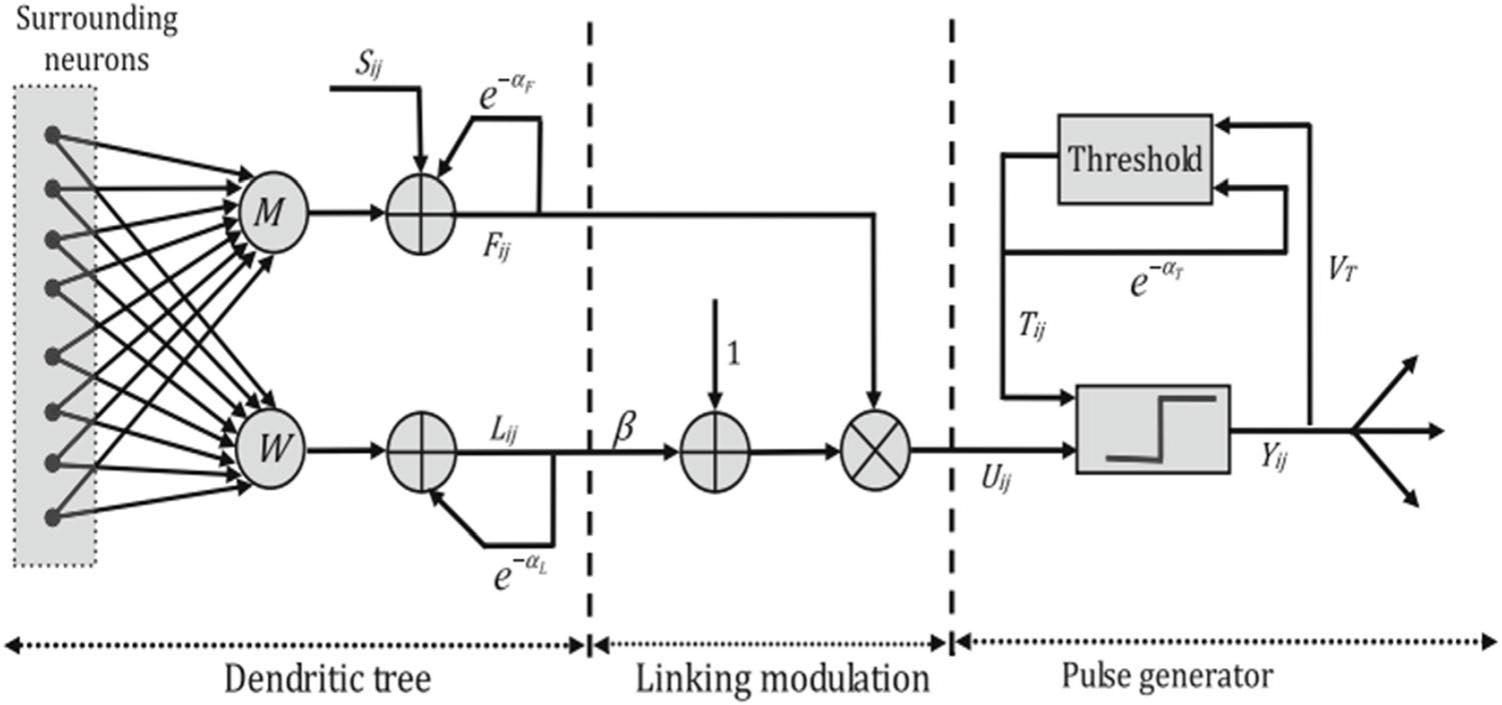
Architecture of the PCNN model[27]

PCNN is the third generation of biological artificial neural network method that is used in many areas such as image processing, object detection, and image fusion. It aims to stimulate and utilize the synchronous pulse emission from the visual cortex for some mammals such as the cat’s brain established in 1990 by Eckhorn et al. [5, 28]. The main benefit of the PCNN method is applied image fusion without a training process [8]. The PCNN is represented as a one-layer network that involves multiple neurons connecting. The following Fig. 3 describes the main PCNN structure. This structure consists of three parts: a dendritic tree, linking modulation, and a pulse generator.

The inputs from the receptive fields are received from the dendritic tree. There are two types of receptive fields. The receptive field types consist of two branches named the linking and the feeding [26]. The role of linking is to receive an external stimulus; on the other hand, the feeding receives both local and external stimulus. The PCNN model can be described mathematically by the following equations [29]:

1 $$\begin{aligned} f_{ij}(n)= & {} e^{-\alpha _{f}}f_{ij}(n-1)+v_{f}\sum \limits _{kl}m_{ijkl}y_{kl}(n-1)+s_{ij}\end{aligned}$$

2 $$\begin{aligned} l_{ij}(n)= & {} e^{-\alpha _{l}}f_{ij}(n-1)+v_{l}\sum \limits _{kl}w_{ijkl}y_{kl}(n-1)\end{aligned}$$

3 $$\begin{aligned} u_{ij}(n)= & {} f_{ij}(n)(1+\beta l_{ij}(n))\end{aligned}$$

4 $$\begin{aligned} y_{ij}(n)= & {} {\left\{ \begin{array}{ll} 1, \ u_{ij}(n)>h_{ij}(n-1)\\ 0, \ otherwise \end{array}\right. }\end{aligned}$$

5 $$\begin{aligned} h_{ij}(n)= & {} e^{-\alpha _{h}}h_{ij}(n-1)+v_{h}y_{ij}(n-1) \end{aligned}$$

where the input channels in the PCNN model are represented by $f_{ij}$(feeding channel) and $l_{ij}$(linking channel) in the *i* and *j* position. The external stimulus is defined by $s_{ij}$, and $m_{ijkl}$ and $w_{ijkl}$ are considered as the local matrix. The neuron’s output is defined by $y_{ij}$. The $\alpha _{f}$, $\alpha _{l}$, and $\alpha _{h}$ are represented as the time constants. The linking coefficient is $\beta$. The voltage is represented by $v_{f}$, $v_{l}$, and $v_{h}$.

## Proposed algorithm

**Fig. 4:**
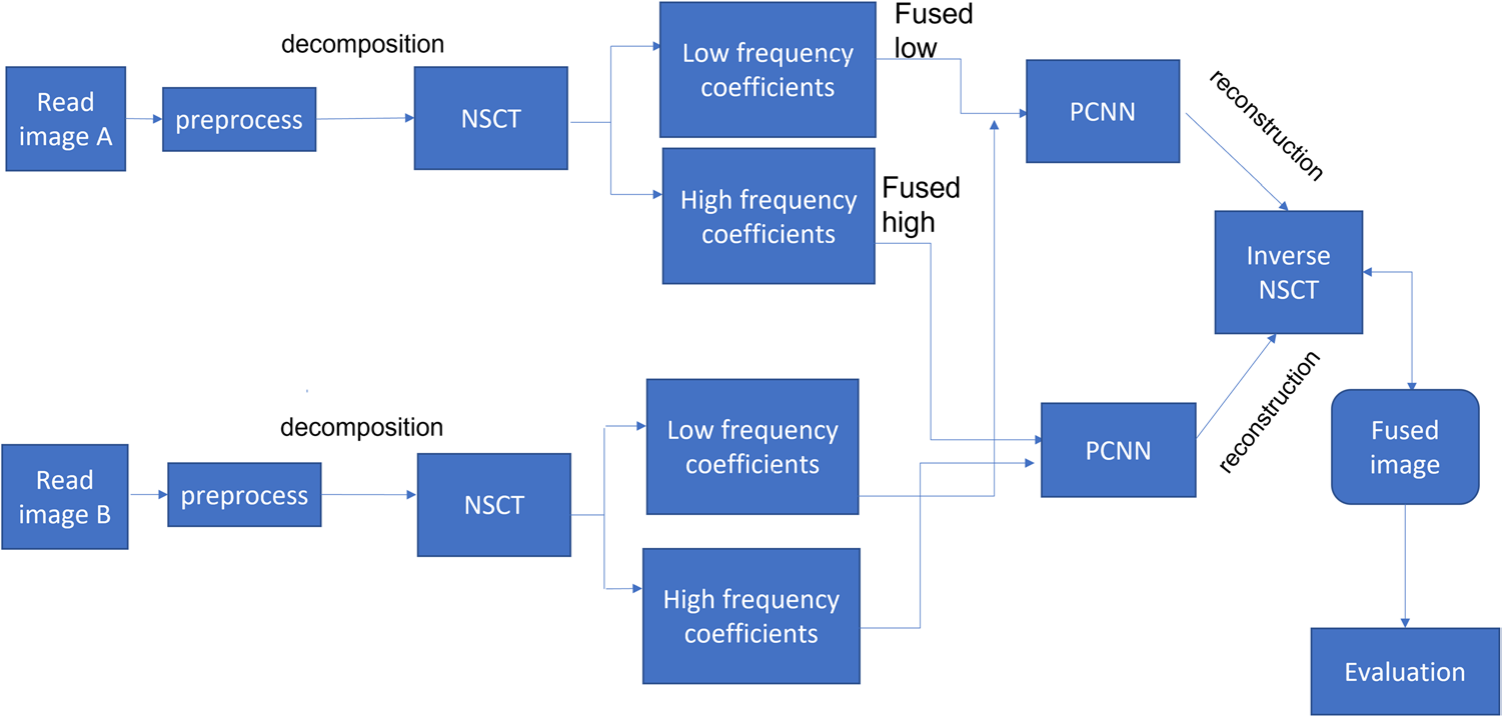
The schematic diagram of the proposed fusion algorithm

In this paper, a multi-modality medical image fusion algorithm is proposed. The proposed algorithm is divided into three basic steps, namely image decomposition, fusion rule, and image reconstruction as shown in Fig. 4.

### Image decomposition

Image decomposition is considered the first step in the proposed algorithm. In this step, we use the NSCT method to decompose preprocessed images A and B into low- and high-frequency subbands $L_{A}$, $H_{A}$, $L_{B}$, and $H_{B}$. The $L_{A}$ is a low-frequency subband for image A, and the high-frequency subband is $H_{A}$. The $L_{B} ~and~ H_{B}$ have the same meaning as image A.

### Fusion rule

Fusion of low- and high-frequency subbands applying the PCNN method as in Eqs. 1 to 4 and calculating the firing time as in Eq. 5, the fused low-and high-frequency coefficients $L_{F}$ and $H_{F}$ are calculated using the following equations:

6 $$\begin{aligned} L_{F}(i,j)= & {} {\left\{ \begin{array}{ll} L_{A}(i,j), \ if \ h_{A,ij}[N]>h_{B,ij}[N] \\ L_{B}(i,j), \ otherwise \end{array}\right. } \end{aligned}$$

7 $$\begin{aligned} H_{F}(i,j)= & {} {\left\{ \begin{array}{ll} H_{A}(i,j), \ if \ h_{A,ij}[N]>h_{B,ij}[N] \\ H_{B}(i,j), \ otherwise \end{array}\right. } \end{aligned}$$

where *N* represents the total number of iterations.

### Image reconstruction

In the NSCT reconstruction step, we use the inverse of the NSCT transform to combine the fused low- and high-frequency coefficients $L_{F}$ and $H_{F}$ to produce the fused image F. Algorithm 2 discusses the steps of the proposed fusion method for multi-modality medical source images.

**Figure:**
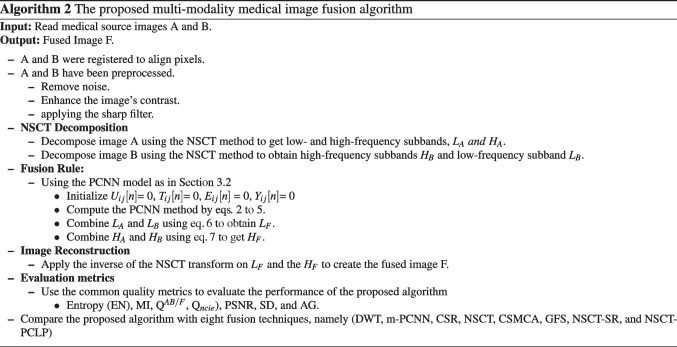
**Algorithm 2** The proposed multi-modality medical image fusion algorithm.

## Experiment results and discussion

In this section, we discuss the details about the results that are used in this paper. This section is divided into four subsections: Datasets, Quality measures, Performance evaluation, and Comparing with other techniques.

### Datasets

In our experiments, these source images are collected from the Whole Brain Atlas database [11]. This database includes both CT and MRI images. We evaluate the proposed algorithm performance by using three pairs of multi-modal medical images. We use 100 pairs of multimodality medical images, 25 image pairs for CT-MRI fusion, 25 image pairs for T1-T2 weighted MRI fusion, and 25 image pairs for CT, MR-PD, and MR-Gad images for normal or abnormal brain diseases. We also use the 25 image pairs for MR-T2, SPECT, and PET images.

All of these images are accurately registered and have the same size of 256*256 pixels. We also use the Matlab R20l8a toolbox to obtain the results. Our experiments are tested on the device with Windows 10, one TB hard disk, 8 GB memory, and an Intel Core i7 processor. Samples of datasets are used in this experiment shown in Table 2.

### Quality measures

In this subsection, we present some evaluation metrics for medical image fusion. There are common evaluation metrics for image fusion. Our experiments use these fusion metrics to evaluate the performance of the proposed algorithm. They are entropy (EN), mutual information (MI), Q$^{AB/F}$, nonlinear correlation information entropy (Q$_{ncie}$), peak signal to noise ratio (PSNR), standard deviation (SD), and average gradient (AG). All of these metrics are discussed as follows.

- **Entropy (EN):** It is useful for measuring the amount of information in the fused image. High EN value means the fused images with high quality and high performance. It is defined as follows:
  8 $$\begin{aligned} EN=-\sum \limits _{l=0}^{L-1}p_{l}\log _{2}p_{l} \end{aligned}$$
  where $p_{l}$ is the ratio of pixels with the gray levels of *l* and *L* represents a total number of gray levels of an image [10, 24].

- **Mutual information (MI): **this metric is used to evaluate the whole information in the fused image. It also measures the relevance or the dependence degree between two or more images [1, 10, 24]. MI is given by:
  9 $$\begin{aligned} MI=MI_{AF}+MI_{BF} \end{aligned}$$
  where *A* and *B* represent the source images and the fused image is represented by *F*. The high MI value means the high-performance fused image. $MI_{AF}$ represents the mutual information between both the source image *A* and the fused image *F*. $p_{A,F}(m,n)$ represent the joint probability of the source and the fused image.
  10 $$\begin{aligned} MI_{AF}=\sum \limits _{m,n}p_{A,F}(m,n)\log _{2}\left[ \frac{p_{A,F}(m,n)}{p_{A}(m)p_{F}(n)}\right] \end{aligned}$$

- **Weighted edge information** (Q$$^{AB/F})$$: total information transferred and edge intensity information from source images to the fused image, which is given as [1, 24]:
  11 $$\begin{aligned} Q^{AB/F}=\tfrac{{\sum _{m=1}^{M}}{\sum _{n=1}^{N}}\left( Q^{AF}(m,n){W_{A}}(m,n)+Q^{BF}(m,n)W_{B}(m,n)\right) }{\sum _{m=1}^{M}\sum _{n=1}^{N}(W_{A}(m,n)+W_{B}(m,n))} \end{aligned}$$
  where the preservation factors of the edge information are denoted by $Q^{AF}$ and $Q^{BF}$, and the weighted items represented by both $W_{A}$ and $W_{B}$. The $Q^{AB/F}$ range is between 0 and 1.

- **Peak signal to noise ratio (PSNR):** one of the main evaluation metrics to measure the quality of the fused image. The high PSNR values represent high-quality images [10] and is given by this equation:
  12 $$\begin{aligned} PSNR=10\log _{10}\left[ \left( 255\right) ^{2}/MSE\right] \end{aligned}$$
  where mean squared error (MSE) is the squared difference between the original image *x*(*l*, *k*) and the output image $\bar{x}(l,k)$ and given by the following equation:

13 $$\begin{aligned} MSE=\frac{1}{MN}\sum _{l=1}^{M}\sum _{k=1}^{N}\left( x(l,k)-\bar{x}(l,k)\right) ^{2} \end{aligned}$$

- **Standarad deviation (SD):** It evaluates the contrast of the fused image by spreading the image data. The high SD value means the fused image with high visibility and good quality image [1, 10]. It is represent by the following equation:
  14 $$\begin{aligned} SD=\sqrt{\frac{\sum _{m=1}^{M}\sum _{n=1}^{N}\left( F(m,n)-\mu \right) ^{2}}{MN}} \end{aligned}$$
  where *MN* represent the size of input image *F*(*m*, *n*) and $\mu$ represent the average of pixel intensity value of the fused image. The $\mu$ is defined as follows:

15 $$\begin{aligned} \mu =\frac{\sum _{m=1}^{M}\sum _{n=1}^{N}F(m,n)}{MN} \end{aligned}$$

- **Average gradient (AG):** the gradient Information of the combined image is evaluated by this metric. It also measures the texture detail such as sharpness and clarity of the fused image [1, 10]. High AG value means the fused image with high performance. The AG metric is given by this equation

16 $$\begin{aligned} {AG}=\sum \nolimits _{m=1}^{M}\sum \nolimits _{n=1}^{N}\sqrt{\frac{\left( (F(m,n)-F(m+1,n))^{2}+(F(m,n)-F(m,n+1))^{2}/2\right) }{MN}} \end{aligned}$$

- **The nonlinear correlation information entropy** Q$_{ncie}$: measures the nonlinear information of the fused image. Q$_{ncie}$ is denoted by the following formula [3]:
  17 $$\begin{aligned} Q_{ncie}(X,Y)=2+\sum \limits _{i=0}^{b^{2}}\left( \frac{n_{i}}{N}\right) \log _{b}\left( \frac{n_{i}}{N}\right) \end{aligned}$$
  where *N* refers to the dataset size and $n_{i}$ refers to the number of samples.

### Performance evaluation

In this subsection, we list some fusion methods used in multimodal image fusion in the medical area. The performance of the proposed algorithm is better if all of these metrics have higher values. We compared the proposed algorithm with eight fusion methods: the discrete wavelet transform (DWT) [13], the multi-channel model–pulse coupled neural networks (MPCNN) [26], the convolutional sparse representation (CSR) [15], the guided image filter and statistics (GFS) [1], the NSCT [13], the convolutional sparsity-based morphological component analysis (CSMCA) [16], the nonsubsampled contourlet transform–sparse representation (NSCT-SR) [17], and the nonsubsampled contourlet transform–phase congruency local Laplacian (NSCT-PCLP) [32].

The parameters in the proposed method are the following: In NSCT, the decomposition level is set 4; “pyrexc” and “vk” are selected. In PCNN, there are too parameters like $\beta ,\alpha _{L},V_{L},\alpha _{\theta },V_{\theta }$ Link_arrange, and number of iterations. The following table describes these parameters (Table 2).

**Table 2:** Some parameters of PCNN

Parameter	Link_arrange	$\beta$	$\alpha _{L}$	$V_{L}$	$\alpha _{\theta }$	$V_{\theta }$	No. of iteration
Values	3	3	1	1	.8	20	100

The bold values represent the PCNN parameter used in our experiment

### Comparing with other techniques

**Fig. 5:**
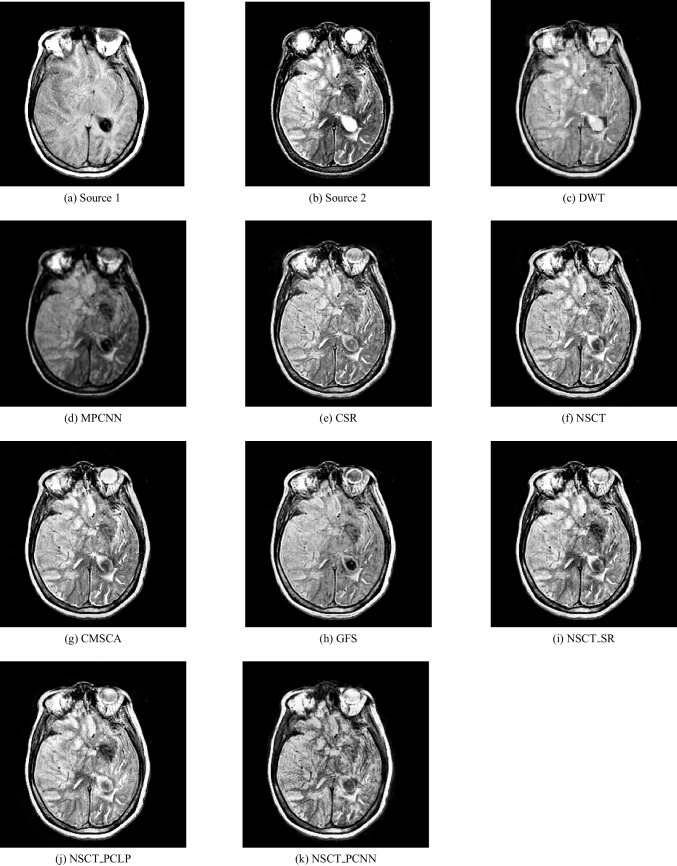
MR-T1/MR-T2 image fusion results

**Fig. 6:**
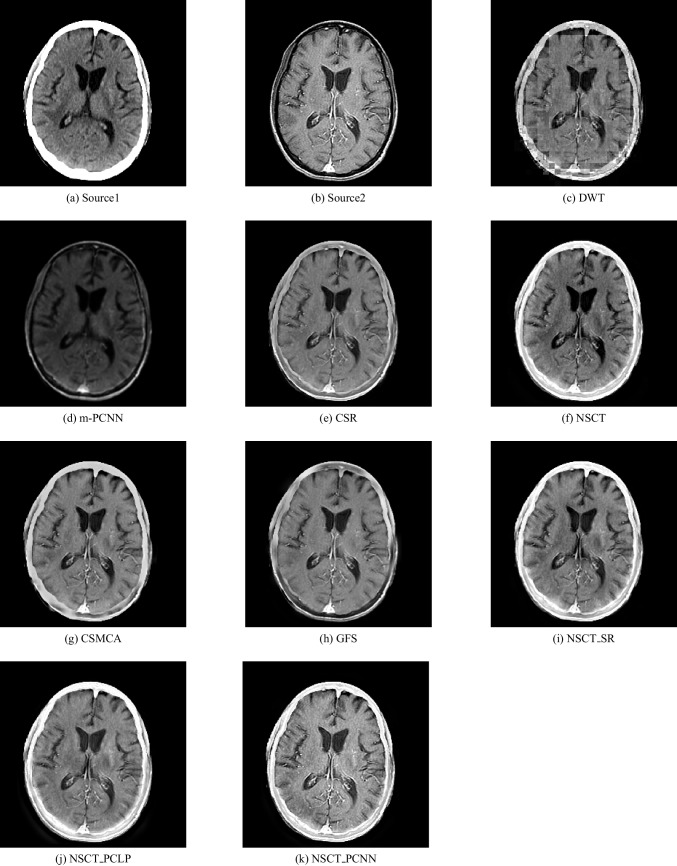
Fusion result of (**a**) MR-T1 and (**b**) MR-T2 using (**c**) DWT method, (**d**) m-PCNN method, (**e**) CSR method, (**f**) NSCT method, (**g**) CSMCA method, (**h**) GFS method, (**i**) NSCT-SR method, (**j**) NSCT-PCLP method, and the proposed method (**k**) NSCT-PCNN

**Fig. 7:**
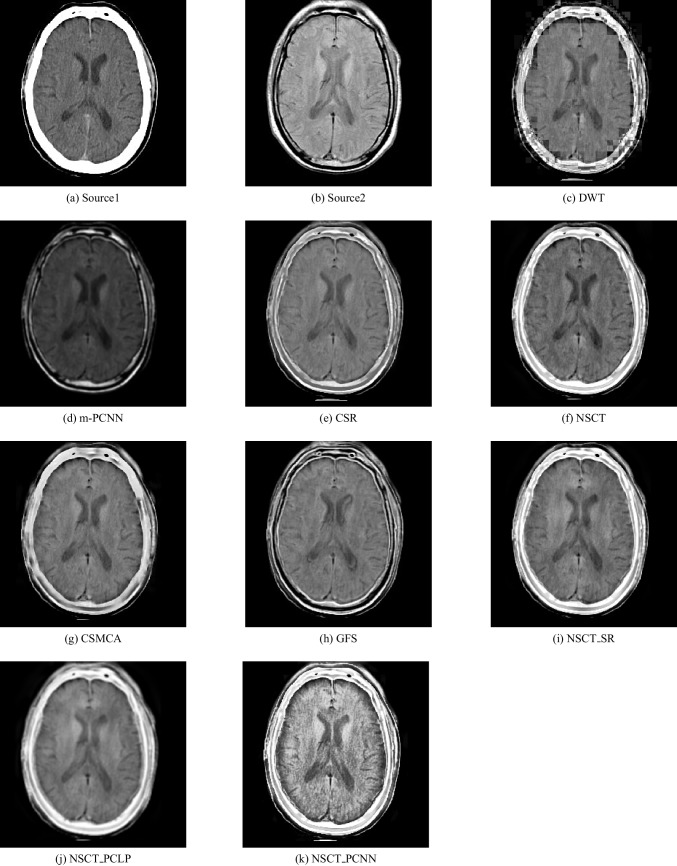
Fusion result of (**a**) CT and (**b**) MR-Gad using (**c**) DWT method, (**d**) m-PCNN method, (**e**) CSR method, (**f**) NSCT method, (**g**) CSMCA method, (**h**) GFS method, (**i**) NSCT-SR method, (**j**) NSCT-PCLP method, and the proposed method (**k**) NSCT-PCNN

**Fig. 8:**
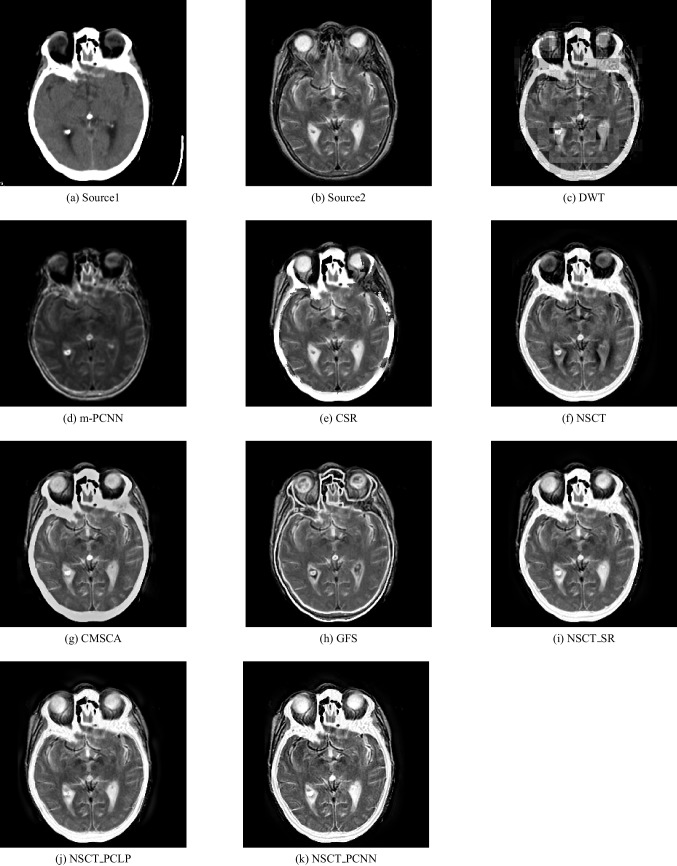
Fusion result of (**a**) CT and (**b**) MR-PD using (**c**) DWT method, (**d**) m-PCNN method, (**e**) CSR method, (**f**) NSCT method, (**g**) CSMCA method, (**h**) GFS method, (**i**) NSCT-SR method, (**j**) NSCT-PCLP method, and the proposed method (**k**) NSCT-PCNN

**Fig. 9:**
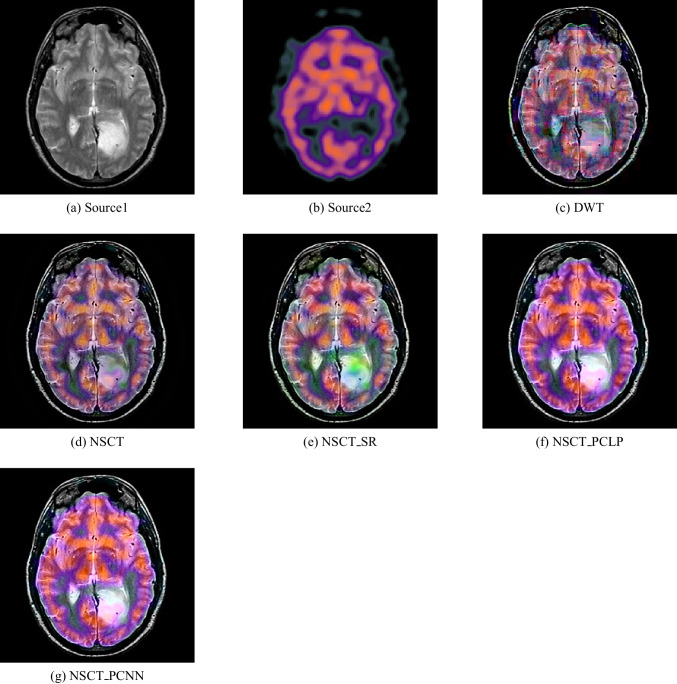
Fusion result of (**a**) CT and (**b**) MR-T2 using (**c**) DWT method, (**d**) m-PCNN method, (**e**) CSR method, (**f**) NSCT method, (**g**) CSMCA method, (**h**) GFS method, (**i**) NSCT-SR method, (**j**) NSCT-PCLP method, and the proposed method (**k**) NSCT-PCNN

**Fig. 10:**
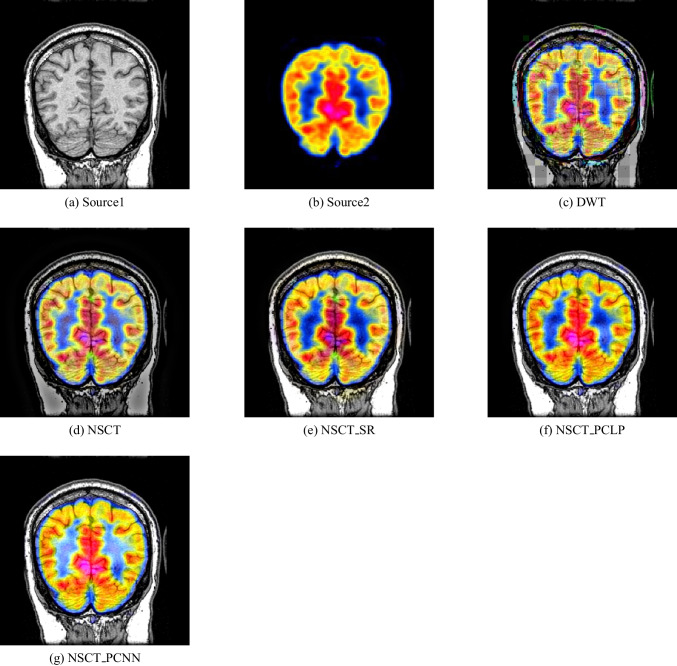
Fusion result of (**a**) MR-T2 and (**b**) SPECT using (**c**) DWT method, (**d**) NSCT method, (**e**) NSCT-SR method, (**f**) NSCT-PCLP method, and the proposed method (**g**) NSCT-PCNN

In our experiments, we apply the proposed algorithm on gray images of four pairs of multi-modal medical images including the following: MR-T1 and MR-T2 images, CT and MR-Gad images, CT and MR-PD images, and CT and MR-T2 images. The following Figs. 5, 6, 7, 8, 9, and 10 show the experiments and results of the proposed algorithm.

Figure 5a is an MR-T1 image and Fig. 5b is an MR-T2 image. In this figure, the fused images of DWT, MPCNN, CSR, NSCT, CSMCA, GFS, NSCT-SR, and NSCT-PCLP are displayed in Fig. 5c, d, e, f, g, h, i, j respectively. The image Fig. 5k represents the fused result of the proposed algorithm. The results show that the DWT and MPCNN methods lose some detailed information from the input image in MR-T2 modality and low contrast images as shown in Fig. 5c and d.

The fused images using the CSR method, the NSCT method, and the CMSCA method represented in Fig. 5e, f, and g are better than Fig. 5c, and d but some detailed information was not detected accurately. In Fig. 5h represented the fused image using the GFS method is good for detecting all image information in Fig. 5a but loses more information from the image in MR-T2 modality. Figure 5i represents the NSCT-SR fused image is detecting more edges and gradient information than Fig. 5j. Figure 5k is the proposed algorithm result with high contrast that preserves both MR-T1 and MR-T2 modality information and prevents visual artifacts.

In Fig. 6a, it is a CT image and Fig. 6b is an MR-Gad image. The results show that Fig. 6c, d, and e lose some detailed information from the input images and produce low contrast images. The results of using the CSMCA method and the GFS method as in Fig. 6g and h visually look good than using the DWT method, MPCNN method, and the CSR method as in Fig. 6c, d, e respectively but do not detect all edges in MR-Gad image. The result of the NSCT-SR method in Fig. 6i is better to fuse CT and MR-Gad images than using the NSCT method and the NSCT-PCLP method. Figure 6k is the fused image of the proposed algorithm with high performance and high contrast, and preserves both CT and MR-Gad modality information without preview visual artifacts.

In Fig. 7a, it is a CT image and Fig. 7b is an MR-PD image. The fused image of the proposed algorithm in Fig. 7k is a high-performance image that contains more mutual information from the input images than using the NSCT-SR method and the NSCT-PCLP method as shown in Fig. 7i and j. In Fig. 8a, it is a CT image and Fig. 8b is an MR-T2 image. The results show that the proposed algorithm in Fig. 8k accurately fused the CT and MR-T2 images and produced high contrast images without preview visual artifacts.

Figure 9 shows the fusion results for MR-T2 and SPECT images. Figure 9a is an MR-T2 image, and Fig. 9b is a SPECT image. The fusion results from the DWT, NSCT, NSCT-SR, and NSCT-PCLP methods perform well in extraction details from MR-T2 images but still have color distortion problems as well as the brain edges cannot detect successfully in Fig. 9c, d, e, and f. The proposed method can preserve color information and achieve higher quality than other methods; see Fig. 9g. Figure 9g shows that the proposed method performs better than NSCT-PCLP as in Fig. 9f on extraction details in some regions.

Figure 10 shows the fusion results for MR-T1 and PET images. Figure 10a is an MR-T1 image, and Fig. 10b is a PET image. The fusion results from the DWT, NSCT, and NSCT-SR can preserve the detailed MR-T1 information with the color fidelity problem in Fig. 10c, d, and e. Figure 10f is better than Fig. 10e in the color fidelity issue but loses some details from the MR-T1 image. The NSCT-PCLP can preserve functional information from the PET image, but some edge and structure information cannot be detected accurately; see Fig. 10f. In Fig. 10g, the proposed method can preserve color and structure information from the source images and achieve higher quality images than other methods.

Tables 3, 4, 5, 6, 7, and 8 report the performance evaluation results of the proposed algorithm and the compared methods. The performance evaluation metrics are calculated, and the highest values at each row shown in bold text are the best score values over all the different used methods. It shows that the proposed NSCT-PCNN algorithm effectively fused medical images and produced high-performance images as compared with other methods. The following figures displayed the values of fusion metrics applied to six pairs of multi-modal medical images, including the following: MR-T1 and MR-T2 images, CT and MR-Gad images, CT and MR-PD images, CT and MR-T2 images, MR-T2 and SPECT images, and MR-T1 and PET images.

**Table 3:** Assessment of different fusion methods on MR-T1/MR-T2 images

Method and metrics	DWT	MPCNN	CSR	NSCT	CSMCA	GFS	NSCT_SR	NSCT_PCLP	Proposed
EN	4.5754	4.3219	4.4240	4.7330	4.3413	4.6281	4.7036	4.8471	**4.9101**
MI	3.3859	3.0288	3.1392	3.1458	3.2119	3.4642	3.2056	3.1677	**3.5121**
Q$^{AB/F}$	0.5472	0.2319	0.5830	0.5817	0.5845	0.5644	0.5820	0.5433	**0.5861**
PSNR	64.4770	61.9325	65.4384	65.1782	64.5221	64.8578	64.6402	64.4505	**66.1580**
SD	80.4108	52.8049	82.4334	85.0782	81.9448	82.5296	85.4476	88.0566	**88.2779**
AG	8.80906	5.40107	12.0605	12.1523	12.1540	11.3904	12.3337	12.0567	**12.5236**
Time (sec)	**3.0480**	33.1315	59.9784	3.1754	1381.08	3.6158	33.6265	3.2978	90.4381

The bold values indicate the best result of the evaluation criterion

**Table 4:** Assessment of different fusion methods on CT/MR-Gad images

Method and metrics	DWT	MPCNN	CSR	NSCT	CSMCA	GFS	NSCT_SR	NSCT_PCLP	Proposed
EN	4.6619	4.0622	4.5380	4.8419	4.5102	4.6686	4.7651	4.8893	**4.9821**
MI	2.9486	2.6828	2.9653	3.1088	3.0709	3.0124	3.1098	3.0928	**3.1953**
Q$^{AB/F}$	0.3715	0.1279	0.4317	0.4152	0.4443	0.4497	0.4291	0.4080	**0.4736**
PSNR	64.4828	60.0427	64.8071	64.6118	64.8093	64.1848	64.7035	64.6530	**64.9061**
SD	69.8985	30.6469	64.5089	76.7335	70.8491	66.0041	78.0360	79.3871	**84.4695**
AG	8.0279	3.2498	6.6523	7.4735	6.6833	6.7030	7.4143	7.3386	**9.6266**
Time (sec)	0.4067	34.7309	55.5042	3.0921	1755.09	**0.3146**	32.9427	2.6856	101.439

The bold values indicate the best result of the evaluation criterion

**Table 5:** Assessment of different fusion methods on CT/MR-PD images

Method and metrics	DWT	MPCNN	CSR	NSCT	CSMCA	GFS	NSCT_SR	NSCT_PCLP	Proposed
EN	5.1882	4.5606	5.0707	5.2326	5.2264	5.0675	5.2382	5.3630	**5.3986**
MI	3.0102	2.8981	3.1521	3.2376	3.1540	3.1720	3.1314	2.9770	**3.2543**
Q$^{AB/F}$	0.3285	0.1949	0.4450	0.3787	0.4033	0.4591	0.3969	0.3244	**0.4704**
PSNR	62.8892	59.1924	62.9195	63.4793	63.2778	61.9589	63.4066	62.4729	**63.9196**
SD	72.5167	36.8359	66.2284	75.6873	70.2274	63.8462	76.9269	81.5905	**85.0497**
AG	8.5255	4.2299	7.3163	7.3904	6.6782	8.8754	7.3899	8.4993	**9.4748**
Time (sec)	0.3183	29.6324	51.4395	1.2437	327.855	**0.2523**	11.4953	2.5246	35.5117

The bold values indicate the best result of the evaluation criterion

**Table 6:** Assessment of different fusion methods on CT/MR-T2 images

Method and Metrics	DWT	MPCNN	CSR	NSCT	CSMCA	GFS	NSCT_SR	NSCT_PCLP	Proposed
EN	4.6183	4.1225	4.6325	4.8642	4.6938	4.6336	4.8684	4.9903	**5.0649**
MI	2.7317	2.4886	2.8179	2.7892	2.9445	2.7919	2.7294	2.9201	**2.9673**
Q$^{AB/F}$	0.3889	0.1880	0.4571	0.4780	0.4908	0.4986	0.4591	0.4280	**0.5127**
PSNR	63.0474	61.0370	61.8477	63.1047	62.8989	62.4608	63.1206	62.9206	**63.2085**
SD	69.6090	34.0936	75.9801	75.5034	71.2350	64.4672	80.3984	79.6444	**81.7336**
AG	7.8589	4.0245	7.8287	8.6292	6.9688	9.8363	7.6959	7.6707	**9.8877**
Time (sec)	0.3975	128.564	58.2724	3.0669	2023.22	**0.2693**	32.5230	4.2180	52.1087

The bold values indicate the best result of the evaluation criterion

**Table 7:** Assessment of different fusion methods on MR-T2/SPECT images

Method and metrics	DWT	NSCT	NSCT-SR	NSCT-PCLP	Proposed
MI	3.0506	2.9943	3.2353	3.4872	**4.0813**
Q$^{AB/F}$	0.6898	0.7186	0.7339	0.7064	**0.7702**
Q$_{ncie}$	0.8075	0.8073	0.8082	0.809	**0.8118**
SD	66.2309	65.9492	70.9162	78.4080	**80.3603**
AG	10.3300	9.9493	10.3019	10.0249	**10.5105**
Time (sec)	**0.9985**	4.5813	33.8711	6.941724	3.983949

The bold values indicate the best result of the evaluation criterion

**Table 8:** Assessment of different fusion methods on MR-T1/PET images

Method and metrics	DWT	NSCT	NSCT-SR	NSCT-PCLP	Proposed
MI	3.0242	2.8750	3.3302	3.5152	**3.8364**
Q$^{AB/F}$	0.6921	0.6159	0.7463	0.7617	**0.7837**
Q$_{ncie}$	0.8073	0.8069	0.8084	0.8093	**0.8108**
SD	80.0882	78.8085	88.1059	91.2964	**94.3089**
AG	11.2366	10.8065	11.4835	11.7440	**11.7520**
Time (sec)	**2.1533**	3.2737	36.3586	6.4638	4.8907

The bold values indicate the best result of the evaluation criterion

**Fig. 11:**
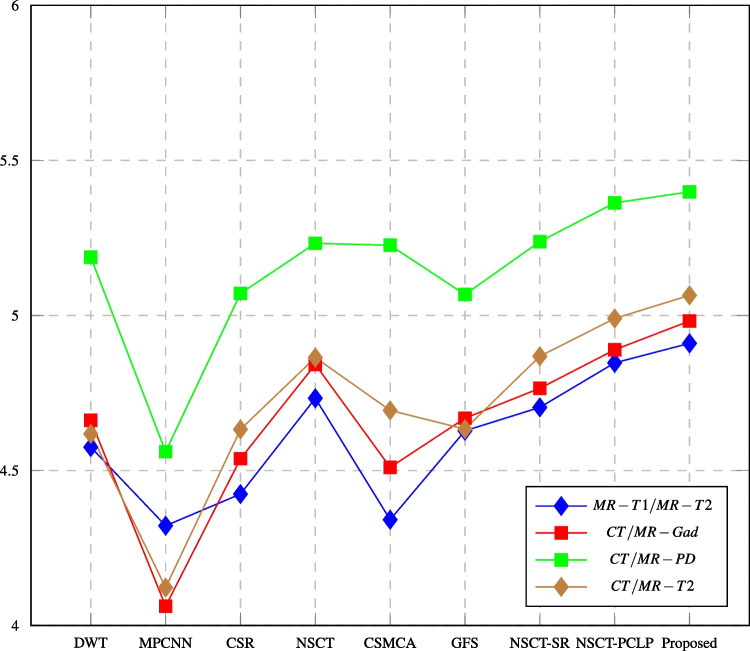
Fusion result of (**a**) MR-T1 and (**b**) PET using (**c**) DWT method, (**d**) NSCT method, (**e**) NSCT-SR method, (**f**) NSCT-PCLP method, and the proposed method (**g**) NSCT-PCNN

**Fig. 12:**
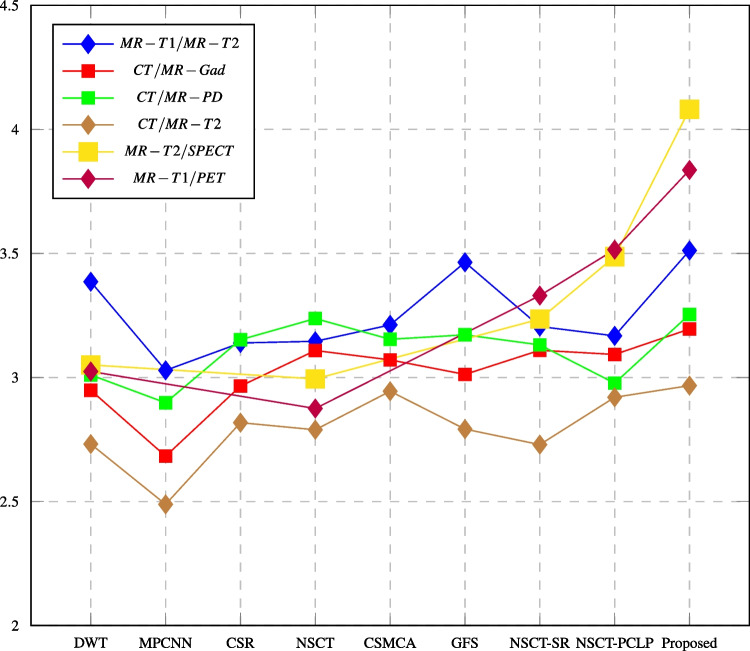
Mutual information assessment of different fusion methods compared to proposed method

**Fig. 13:**
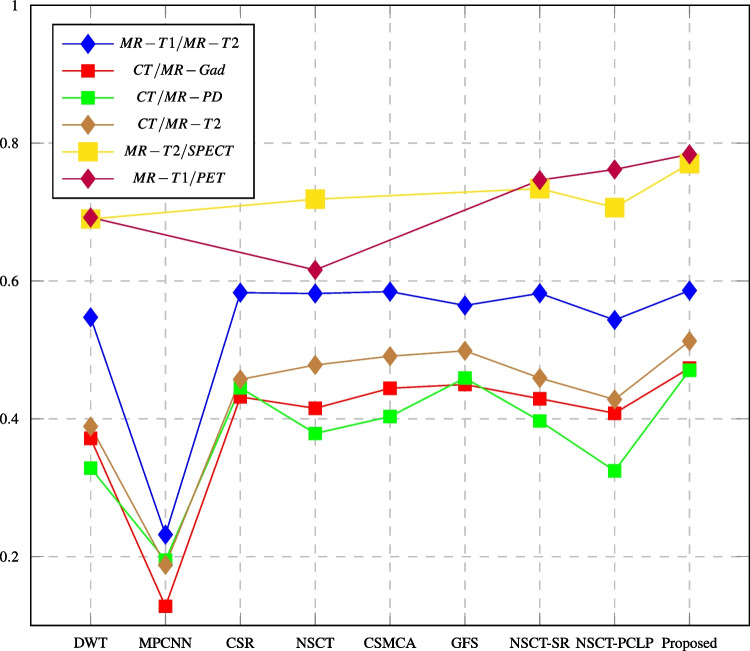
Q$^{AB/F}$ assessment of different fusion methods compared to proposed method

**Fig. 14:**
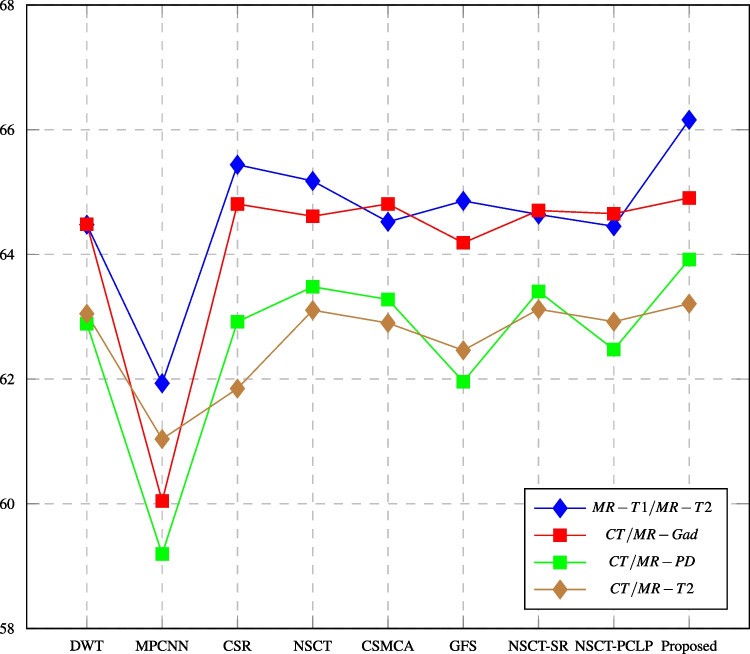
PSNR assessment of different fusion methods compared to proposed method

**Fig. 15:**
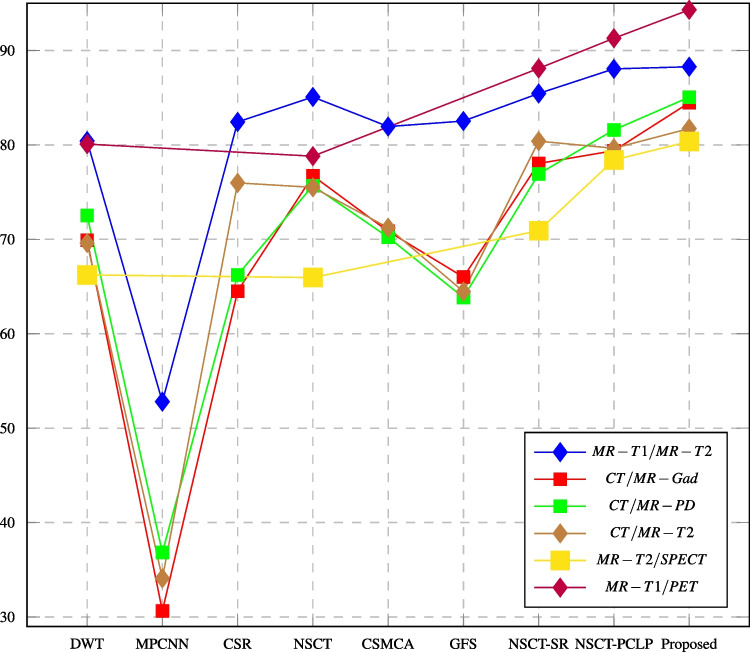
SD assessment of different fusion methods compared to proposed method

**Fig. 16:**
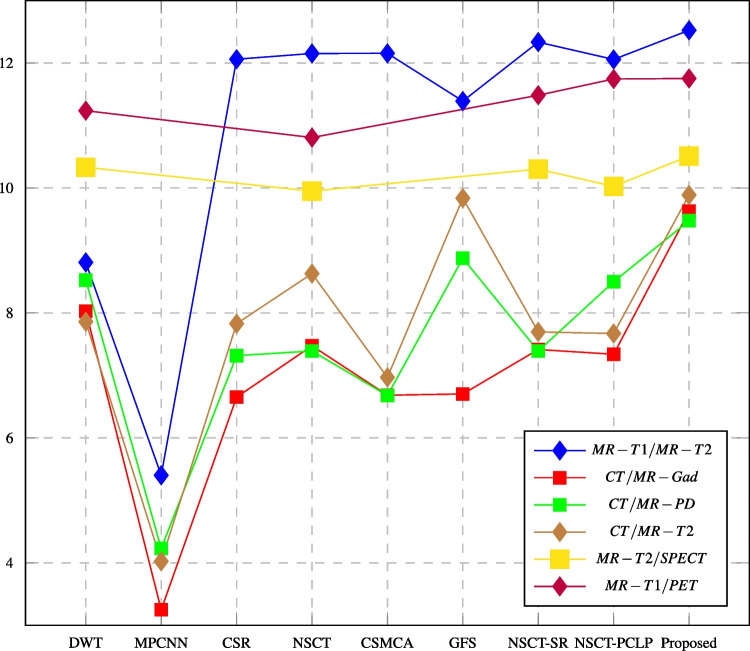
AG assessment of different fusion methods compared to proposed method

**Fig. 17:**
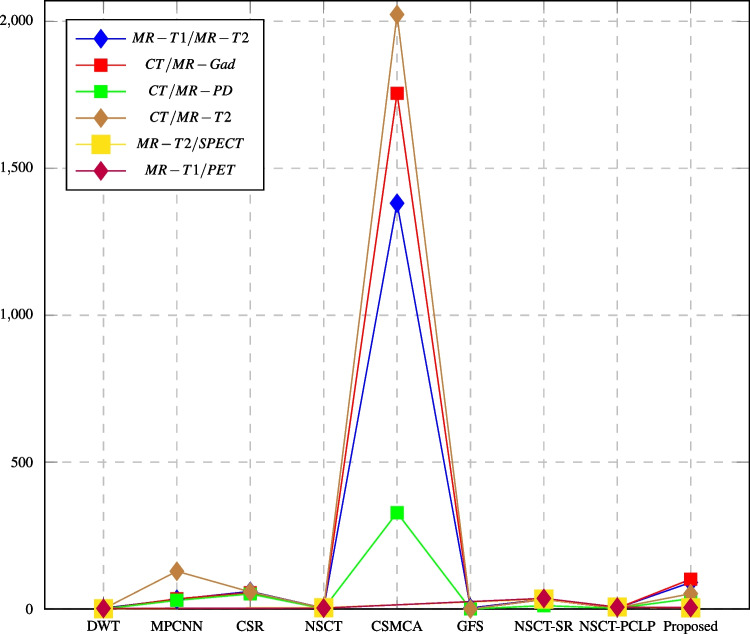
Time assessment of different fusion methods compared to proposed method

**Fig. 18:**
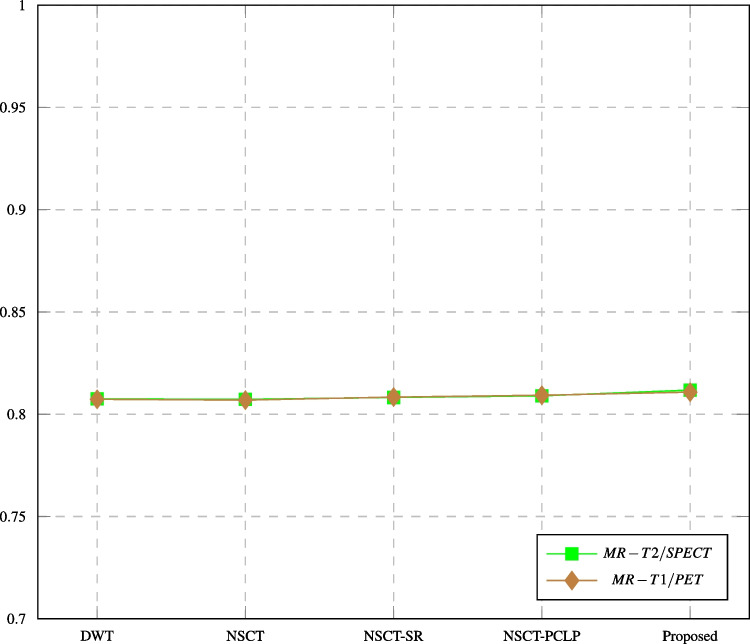
Q$_{ncie}$ assessment of different fusion methods compared to proposed method

Table 7 shows the quantitative and objective assessments of the proposed algorithm and the compared methods on MR-T2/SPECT images. The proposed algorithm is better than other compared methods in MI, Q$^{AB/F}$, Q$_{ncie}$, SD, and AG values. The time in the DWT is better than the proposed algorithm time. Table 8 shows the quantitative and objective assessments of the proposed algorithm and the compared methods on MRT1/PET images. Our proposed algorithm has higher values than other compared methods in MI, Q$^{AB/F}$, Q$_{ncie}$, SD, and AG. The time in the DWT is better than the proposed algorithm time. The results show that the proposed algorithm performs better than other compared methods in both objective and visual quality, retaining more information from the source images.

In this paper, major objective metrics including EN, MI, Q$^{AB/F}$, PSNR, SD, and AG have evaluated the fusion performance for the DWT, MPCNN, CSR, NSCT, CSMCA, NSCT-SR, NSCT-PCLP, and the proposed algorithm using MR-T1/MRT2, CT/MR-GAD, CT/MR-PD, and CT/MR-T2 images. These metrics are represented in Figs. 11, 12, 13, 14, 15, 16, and 17. For MR-T2/SPECT and MR-T1/PET images, the fusion performance for the DWT, NSCT, NSCT-SR, NSCTPCLP, and the proposed algorithm is evaluated in Figs. 12, 13, 15, 16, 17, and 18.

## Conclusion

In this paper, a new multimodal medical image fusion algorithm is proposed. The proposed algorithm is based on the NSCT and PCNN methods. This algorithm is divided into three main steps: decomposition, fusion rule, and reconstruction. First, the NSCT method is applied to decompose two input images from multi-sensors. In this step, the input images are decomposed by the NSCT method into low- and high-frequency subbands. Then, apply the PCNN method as a fusion rule that fuses both the high- and low-frequency subbands. Finally, apply the inverse of the NSCT method to both fused low- and high-frequency subbands and construct the final fused image. Our experiments are implemented on six sets of medical images: MR-T1 and MR-T2 images, CT and MR-Gad images, CT and MR-PD images, CT and MRT2 images, MR-T2 and SPECT images, and MR-T1 and PET images were obtained from the Whole Brain Atlas database. To evaluate the performance of the proposed algorithm, we use common fusion metrics, namely entropy, mutual information, $Q^{AB/F}$, PSNR, standard deviation, Q$_{ncie}$, and average gradient. The experimental results show that the proposed algorithm has high performance as compared with others.

## Abbreviations

AG: Average gradient
CNN: Convolutional neural networks
CSMCA: Convolutional sparsity-based morphological component analysis
CSR: Convolutional sparse representation
CT: Computed tomography
CT: Contourlet transform
DWT: Discrete wavelet transform
EN: Entropy
GB: Giga byte
GFS: Guided image filter and statistics
IPCNN: Improved PCNN
LP: Laplacian pyramid
MI: Mutual information
MPCNN: Multi-channel model–pulse coupled neural networks
MRI: Magnetic resonance imaging
MSE: Mean square error
MST: Multiscale transform
NSCT: Nonsubsampled contourlet transform
NSCT-PCLP: Nonsubsampled contourlet transform–phase congruency local Laplacian
NSCT-SR: Nonsubsampled contourlet transform–sparse representation
NSPFB: Nonsubsampled pyramid filter bank
NSDFB: Nonsubsampled directional filter bank
NSST: Nonsubsampled shearlet transform
PCA: Principal component analysis
PCNN: Pulse coupled neural network
PET: Positron emission tomography
PSNR: Peak signal to noise ratio
RMSE: Root mean square error
SD: Standard deviation
SF: Spatial frequency
SPECT: Single-photon emission computed tomography
SSIM: Structural similarity model
TB: Terabyte
